# Effect of Carbohydrate Intake on Maximal Power Output and Cognitive Performances

**DOI:** 10.3390/sports4040049

**Published:** 2016-10-09

**Authors:** Laura Pomportes, Jeanick Brisswalter, Arnaud Hays, Karen Davranche

**Affiliations:** 1 Université Cote d’Azur, Laboratoire Motricité Humaine Expertise Sport Santé, Nice 06205, France; brisswalter@unice.fr; 2 CREPS PACA, Aix en Provence 13098, France; 3 Aix-Marseille Université, CNRS, ISM, Marseille 13288, France; arnaud.hays@univ-amu.fr; 4 Aix-Marseille Université, CNRS, LPC UMR 7290, Marseille 13331, France

**Keywords:** CHO, maximal muscular performance, information processing

## Abstract

The present study aimed to assess the beneficial effect of acute carbohydrate (7% CHO) intake on muscular and cognitive performances. Seventeen high levels athletes in explosive sports (fencing and squash) participated in a randomized, double-blind study consisting in series of 6 sprints (5s) with a passive recovery (25s) followed by 15 min submaximal cycling after either maltodextrine and fructose (CHO) or placebo (Pl) intake. Cognitive performances were assessed before and after sprint exercise using a simple reaction time (SRT) task at rest, a visual scanning task (VS) and a Go/Nogo task (GNG) during a submaximal cycling exercise. Results showed a beneficial effect of exercise on VS task on both conditions (Pl: −283 ms; CHO: −423 ms) and on SRT only during CHO condition (−26 ms). In the CHO condition, SRT was faster after exercise whereas no effect of exercise was observed in the Pl condition. According to a qualitative statistical method, a most likely and likely positive effect of CHO was respectively observed on peak power (+4%) and tiredness (−23%) when compared to Pl. Furthermore, a very likely positive effect of CHO was observed on SRT (−8%) and a likely positive effect on visual scanning (−6%) and Go/Nogo tasks (−4%) without any change in accuracy. In conclusion acute ingestion of 250 mL of CHO, 60 min and 30 min before exercise, improve peak power output, decrease muscular tiredness and speed up information processing and visual detection without changing accuracy.

## 1. Introduction

Classically, the ergogenic effects of carbohydrate (CHO) ingestion on physical performance have been demonstrated in the field of sports nutrition research. Indeed, as early as 1925, Gordon et al. [1] reported that ingestion of candy by runners during a marathon prevented hypoglycemia and improved race times when compared with no sugar. The “metabolic response” of CHO feeding allows maintaining plasma glucose concentration and high rates of carbohydrate oxidation, sparing hepatic glycogen, delaying muscle glycogen depletion and preventing hypoglycemia [2]. The beneficial effect of CHO supplementation during exercise also seems to affect the central nervous system (CNS) by increasing or maintaining the substrate delivery to the brain and avoiding hypoglycemia, which is known to impair brain function and cognitive performances [3]. Further evidences suggest that CHO may have a “non-metabolic” central effect. According to the “Central Fatigue hypothesis” proposed by Newholme et al. in 1987 [4], tryptophan (which is an essential amino acid and a precursor of serotonin) is associated with central nervous system fatigue during prolonged exercise. It appears that CHO feeding decreases the amount of tryptophan crossing the blood–brain barrier and consequently leads to lower serotonin concentrations in the brain [3,5]. 

The positive effect of CHO feedings on performance has been mainly observed during prolonged exercise (>1 h) (for review see [6]). Endurance exercise has been associated with a decrease in CHO availability that includes significant glycogen reduction, low blood glucose, and elevations in free fatty acids. These phenomena can be avoided by CHO feedings. In intermittent sports that required brief high-intermittent exercise, more mixed results have been reported. Most studies report an enhancement of intermittent high-intensity exercise capacity (measured by the duration to fatigue and distance covered) with CHO supplementation before and during exercise (for review see [7]). Mixed results are reported on other aspects of intermittent sport performance such as sprinting, jumping, skill, change of direction speed, and cognitive function (for review see [8]). For short duration exercise the effect of CHO feeding is unlikely explained by a metabolic response but more by an ergogenic effect highlighted using mouth rinsing. In a study employing a Loughborough Intermittent Shuttle Test (LIST) protocol (mimicking the demands of soccer and similar team sports), Dorlin and Earnest [9] found no effect of mouth rinsing 6.4% maltodextrin, in comparison with a mouth rinsing placebo. By contrast, using a self-selected pacing LIST protocol, Rollo et al. [10] reported improved sprint performance with mouth rinsing 10% maltodextrin compared to a placebo. It is likely that CHO mouth rinsing exerts a positive effect through a central action mediated by receptors in the mouth or gastro-intestinal tract, improving motor drive or motivation [11,12,13,14]. Gant et al. [11] first showed that CHO in the mouth immediately increases the excitability of the cortico-motor pathway, prior to ingestion. More recently, using a qualitative approach, De Pauw et al. [12] showed an increased activity within the orbitofrontal cortex during maltodextrine mouth rinse, but did not report any change on response speed in a Stroop task. The improvement in exercise performance observed when CHO is present in the mouth can be explained by an activation of regions involved in reward and motor control (primary taste cortex and dorsolateral prefrontal cortex) [13] and by increasing the primary sensorimotor cortex, which enhances activation of neural networks involved in sensory perception [14]. The effect of CHO ingestion on sprint performance, however, remains unclear. In a review on this topic, Baker et al. indicate that most of the studies report no benefit of CHO ingestion on sprint performance [8]. However some of them indicate that CHO ingestion could improve performance. For example, Gant et al. [11] reported enhanced sprint performance during a 60-min LIST protocol when soccer players ingest a 6.2% CHO solution, and Ali et al. [15] reported similar benefit with a 6.4% CHO solution during a 90-min LIST protocol. Additionally, Lee et al. [16] showed that CHO intake prior to exercise provided a significant benefit on repetition sprint performance in female athletes. Finally, Welsh et al. [17] and Winnick et al. [18] reported improvement in sprint performance when basketball players respectively intake 80 g of carbohydrate per hour (g/h) and 40 g/h. 

Intermittent court sports like squash or fencing are characterized by intermittent bursts of high-intensity exercise and require the execution of complex sport-specific skills and cognitive tasks over a prolonged period of time of several times per day [8]. Athletes have to simultaneously perform very intense short exercises and make decisions under strong temporal pressure. The physiological effects of exercise on cognitive performance have been well documented [19,20]. According to the different meta-analyses on exercise and cognition, the presence or absence of a beneficial effect of exercise on cognitive performance depends on the nature of the cognitive task but also on exercise intensity and duration [21]. A positive effect of acute moderate exercise is generally explained by an activation of the CNS, with the assumption that exercise-induced physical arousal leads to a narrowing of attentional focus [22], whereas a heavy exercise or prolonged exercise to exhaustion leads to a decrease in cognitive performance [23]. Within this framework, it has been suggested that nutritional supplementation could help to limit central fatigue and increase or maintain cognitive abilities [24]. For example, cognitive performance improvement has been observed during exercise when CHO was ingested before or during exercise [22,25,26,27]. Collardeau et al. 2001 [22] showed that consumption of a 5.5% CHO electrolyte solution before and during a 100-min run improved the reaction time (RT) of triathletes at the end of exercise. Bottoms et al. [25] also reported that ingestion of a 6.4% CHO drink improved RT performances during a simulated squash play. Lieberman et al. [26] found that supplemental CHO beverages (12%) enhance vigilance and mood during sustained aerobic exercise (road march and run), and Utter et al. [27] reported that CHO availability attenuates exertion perceptions during the last stages of 2.5 h high-intensity cycling and running exercise. 

The current state of the art showed that CHO feedings could improve both physical and cognitive performances; however, the effect of CHO supplementation on cognitive functions has been essentially assessed during endurance exercises. The present study aimed to investigate the effect of CHO supplementation on intermittent sport performance, more particularly on sprint and cognitive performances, during a protocol involving brief high-intermittent exercise in high-level athletes in intermittent sports (e.g., squash and fencing).

## 2. Methods

### 2.1. Participants

Seventeen high-level squash and fencing athletes (Table 1) (7 females and 10 males) volunteered in the study. Participants were used to training on a cycle ergometer and cycling as a normal part of their daily training program. The experimental procedures were explained to the subjects and a written informed consent was provided and signed prior to inclusion. The present study was approved by the University Ethics Committee.

### 2.2. Design

#### 2.2.1. Preliminary Session

One week before the experimental sessions, a preliminary testing was performed to collect anthropometric and physiological characteristics. Body weight was measured to 0.1 kg using a standard beam balance. Height was measured to 0.5 cm using a Holtain wall mounted stadiometer. In all subjects, four skinfold thickness (biceps, triceps, subscapular, suprailiac) were measured in triplicate, by the same trained observer. Measurements were made on the right-hand side of the body using a Holtain caliper. Body fat was calculated using the Siri equation. Maximal oxygen uptake (VO_2max_) was determined during a field maximal incremental exercise according to the protocol proposed by Léger and Boucher [28]. The speed of the last fully completed step corresponds to the maximal aerobic speed. During the test, oxygen uptake (VO_2_), carbon dioxide uptake (VCO_2_), minute ventilation (VE) and the respiratory exchange ratio (RER) were continuously recorded using a breath-by-breath analyzer (K4b2, Cosmed^®^, Rome, Italy) which was calibrated prior to each test. Heart rate was collected using a monitor (Polar RS800). Before and 3 min after running sessions, blood lactate concentrations were measured (LactatePro, Arkray Factory Inc., Shiga, Japan). 

The target heart rate of the experimental sessions was determined from a competition simulation respectively in squash (5 players) and fencing (5 players) against a similar level opponent. For the squash simulation, 5 games of 11 points (1 for each players lasting between 8 and 13 min) were recorded and analyzed, whereas for fencing athletes, 5 matches of 3 min were recorded. From these records, the relative intensity was set at 80% of HR_max_ for fencing athletes and 85% FC_max_ for squash athletes. 

#### 2.2.2. Familiarization Session

Seventy-two hours prior to the first experimental session, subjects underwent a familiarization session with the repeated sprint effort protocol on a cycle ergometer and with the following cognitive tasks (CT): simple reaction time (SRT), visual scanning and Go/Nogo tasks (TAP software 2.3 version 3, P. Zimmermann and B. Fimm). For CT, the learning criteria was achieved when response variability was equal or less than 10% compared to the preceding cognitive performance.

#### 2.2.3. Experimental Session

Each subject participated in two counterbalanced experimental sessions separated by 1 week at the same time of the day. Subjects were instructed to keep a food diary during the 2 days prior the first session and to replicate this diet before each session. Subjects were required to refrain from alcohol, caffeine, drugs, and nutritional supplements during the 48 h prior the sessions. The last meal was taken at least 3 h prior the start of the experimental session. No food or drinks intake (except water) was allowed between the last meal and the experimental session. The subjects were not used to regularly drinking nutritional supplementation during training or competitions. They were instructed to control their time and hours of sleep the 2 nights preceding the experimental sessions. Light trainings were permitted in the morning of the experimental sessions whereas physical training (including endurance or resistance training) or high-intensity technical sessions were not allowed. Subjects were required to maintain the same training program every week during the experimental protocol. 

**Figure 1:**
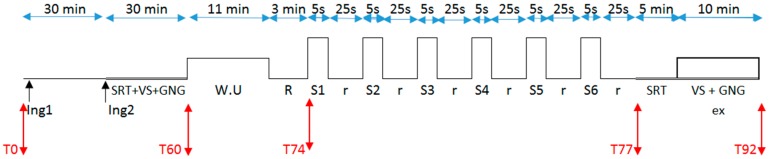
Overview of the experimental design. Notes: Ing = ingestion, W.U = warm up, R = rest 3 min, S1 to S6 = sprints 5 s, r = 25 s rest, SRT = simple reaction time, VS = visual scanning, GNG = Go/Nogo tasks, ex = cycling exercise, T = time (minutes).

During each experimental session, subjects ingested 60 min (Ing1) and 30 min (Ing2) before exercise either 250 mL of a 7% carbohydrate complex (CHO: fructose and maltodextrine, Isoxan Sport,) or placebo (Pl, 250 mL tap water added with orange sugarless syrup). Immediately after the second ingestion subjects performed the first cognitive tests block (i.e., CT). Then, subjects warmed up (standardized warm up) and after 3 min recovery performed, they performed a repeated sprint test in order to assess maximal power output. Immediately after this test, subjects completed one block of SRT. Finally, they performed a 15 min submaximal cycling exercise at 80% and 85% HR_max_, respectively for fencing and squash athletes, during which they performed the visual scanning and Go/Nogo tasks (Figure 1). 

### 2.3. Repeated Sprint Test 

Maximal power output was assessed during the repeated sprint test on a Wattbike cycle (Wattbike Pro, Nottingham, UK). This ergometer has been designed to simulate “real” cycling with a suitable power output range (0 to 3760 watts) for short-duration, high-intensity testing, or training, and has been previously validated to assess sprint performance in cyclists [29]. After a specific warm up for sprint performance, subjects performed 6 × 5 s sprints (S1 to S6), with a 25 s passive recovery between each sprint (the resistance was set at the minimum, resistance 1). Maximal power output was recorded for each sprint, and tiredness (%) was calculated using the difference between maximal power output for the best and the last sprint.

### 2.4. Cognitive Tasks (CT)

During the two experimental sessions, subjects were comfortably seated on the cycle ergometer facing a computer screen 1 meter away. Two joysticks were installed on the ergocycle. Participants were required to complete the CT that consisted in a series of 3 cognitive tasks (i.e., SRT, visual scanning and Go/Nogo tasks) before and after the series of 6 sprints. The SRT was performed at rest immediately after the sprints, and the visual scanning and Go/Nogo tasks were performed while cycling about 15 min at 80% and 85% HRmax respectively for fencing and squash athletes.

#### 2.4.1. Simple Reaction Time (SRT)

Simple reaction time is a perceptual-motor task indicative of the efficiency of the individual’s ability to process information. In the SRT task, subjects were asked to respond as quickly as possible to the visual known stimuli by pressing the button of a joystick with their dominant hand. The SRT task duration was about 3 min 30 s.

#### 2.4.2. Visual Scanning Task

Visual scanning task (TAP 2.3 software) is an attention/perceptual task, during which subjects had to search a display for a given stimulus. The display was a matrix on a row-by-row or column-by-column basis (5 × 5 stimuli). Subjects were instructed to press one key response as soon as possible when they detected the target stimulus, and to press another key response when the target stimulus was absent. The visual scanning task duration was about 5 min.

#### 2.4.3. Go/Nogo Task

The Go/Nogo task evaluates inhibitory control. Subjects were instructed to respond to stimuli and withhold the response to other stimuli. In the present version of the task (TAP 2.3 software), five squares with different patterns appeared on the screen. Two of these squares were defined as target stimuli. Subjects were instructed to press a key response as soon as possible when a target stimulus was presented, and withhold their response when a non-pertinent stimulus appeared. The Go/Nogo task duration was about 3 min. 

### 2.5. Statistical Analysis

Results were analyzed using consecutively quantitative and qualitative methods. All data are expressed as the mean ± standard error. For sprint performance (i.e., mean peak power and tiredness of 6 sprints), difference between conditions was assessed using a paired t-test. For cognitive performances, a repeated measures ANOVA (condition × period) was performed. Post-hoc Newman-Keuls analyses were conducted for all significant effects. Significance was set at p < 0.05 for all analyses. We also reported probabilistic magnitude-based inferences for all variables data using methods described by Hopkins et al. [30], which has been applied in several recent nutritional studies [14]. To compare within-trial changes between trials, we used a modified statistical spreadsheet. This spreadsheet calculates the between-trial standardized differences or effect sizes (ES, 90% confidence interval [CI]) using the pooled standard deviation. If the chance of benefit or harm were both >5% the true effect was reported as unclear. Otherwise, chances of benefit or harm were assessed as follows: <1%, almost certainly not; 1%–5%, very unlikely: 5%–25%, unlikely; 25%–75%, possible; 75%–95%, likely; 95%–99%, very likely; >99%, most likely [5].

## 3. Results

### 3.1. Sprint Performance 

**Figure 2:**
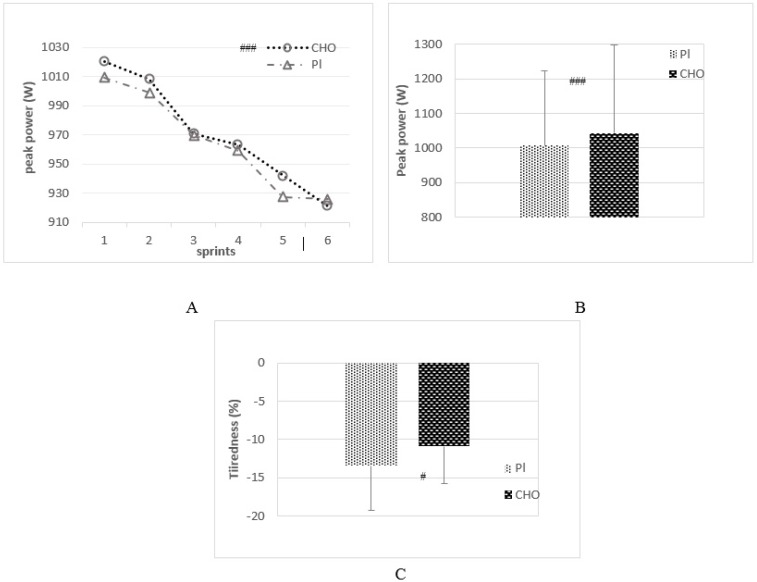
Evolution of peak power with sprint (**A**); peak power (**B**) and tiredness (**C**) with placebo (Pl) and carbohydrate (CHO) ingestion (mean ± standard error). Notes: # when a qualitative statistics shown an effect compared with Pl condition: # likely effect, ## very likely effect and ### most likely effect.

There were no significant differences between conditions (CHO vs. Pl) on peak power and tiredness. Magnitude-based inferences showed that CHO ingestion has a most likely positive effect on peak power (99%, CI: 9.4/48.8 watts) (Figure 2A,B). Mean peak power was 1043 ± 255 watts in CHO and 1007 ± 218 watts in Pl. Furthermore a likely positive effect (77%, CI: 0.8/5.2%) was observed on tiredness (Figure 2C) with a lower decrease of peak power among sprints when compared to Pl (respectively for CHO and Pl: −11% ± 4.9% vs. −13% ± 5.8%).

### 3.2. Cognitive Performance

An overview of the results for cognitive performance is shown on Table 2.

#### 3.2.1. Simple Reaction Time (SRT) 

A significant interaction effect between exercise and condition was observed on mean RT (F_(1,16)_ = 6.95, p = 0.002, η_p_^2^ = 0.30). In the CHO condition, RT was faster after exercise (−11%) compared to pre-exercise and faster than Pl post-exercise (−7.9%). No effect of exercise was observed in the Pl condition (−1%). Magnitude-based inferences showed that the effect of exercise is likely positive (93%, CI: −43.3/8.7 ms) on RT (i.e., faster RT) on both conditions, and the effect of CHO ingestion, compared to Pl, is likely positive (96%, CI: −15.5/3.7 ms) on RT performance decrease after exercise.

No significant effect of nutritional supplementation and/or exercise was found on standard deviation (respectively F_(1,16)_ = 0.11, p = 0.074, η_p_^2^ = 0.007 and F_(1,16)_ = 1.05, p = 0.032, η_p_^2^ = 0.06). Magnitude-based inferences showed that the effect of CHO ingestion, compared to Pl, is likely positive (78% CI: −23/11 ms) on SD before sprints and likely negative (90%, CI: −8/16 ms) on SD (i.e., increase in SD) after sprints indicating a higher variability in response after exercise. 

#### 3.2.2. Visual Scanning

Results showed a main effect of exercise (F_(1,15)_ = 24.78, p = 0.000, η_p_^2^ = 0.62) on RT during visual scanning regardless of the conditions. Reaction time was faster after exercise for Pl (−14%) and CHO (−21%) (p < 0.001). Magnitude-based inferences showed that the effect of exercise is most likely positive (100%, CI: −148/−67 ms) in both conditions. Furthermore, CHO ingestion has a likely positive effect (86%, CI: −12.2/−0.2) on RT when compared to Pl during exercise.

Results showed a main effect of exercise (F_(1,15)_ = 20.58, p = 0.000, η_p_^2^ = 0.58) on SD in both conditions. A significant decrease of the SD was observed after exercise for Pl (−20%) and CHO (−20%) conditions. Magnitude-based inferences showed the effect of exercise is most likely positive (100%, CI: −220/−105 ms) in both conditions. Furthermore, CHO ingestion has a likely positive effect (96%, −117/26 ms) on SD before sprints when compared to Pl. 

No effect of nutritional supplementation and exercise was observed on tiredness (respectively F_(1,15)_ = 0.00, p = 0.098, η_p_^2^ = 0.00 and F_(1,15)_ = 2.72, p = 0.012, η_p_^2^ = 0.15). Magnitude-based inferences indicated that exercise has a likely positive effect (87%, CI: −12.5/0.5%) on tiredness (decrease), regardless of the nutritional supplementation used.

Results showed a main negative effect of exercise on errors (F_(1,16)_ = 5.05, p = 0.004, η_p_^2^ = 0.240) (increase of errors). No effect of nutritional supplementation was observed (F_(1,16)_ = 1.78, p = 0.021, η_p_^2^ = 0.10). Magnitude based inferences showed that the effect of exercise is likely negative (79%, CI: 0.2/3.9%) on accuracy in both conditions whereas CHO ingestion is likely negative (94%, CI: 0.2/2.2%) on accuracy when compared to Pl before sprints.

#### 3.2.3. Go/Nogo

No effect from nutritional supplementation and exercise was observed on mean RT (respectively F_(1,16)_ = 0.26, p = 0.062, η_p_^2^ = 0.02 and F_(1,16)_ = 0.14, p = 0.072, η_p_^2^ = 0.01). Magnitude-based inferences showed that exercise has a likely positive effect (70%, CI: −43.8/8.2 ms). Furthermore, the effect of CHO ingestion is likely positive (82%, CI: −11.8/0.2) on RT compared with Pl condition during exercise. 

No effect from nutritional supplementation and exercise was found on SD (respectively F_(1,16)_ = 0.13, p = 0.072, η_p_^2^ = 0.01 and F_(1,16)_ = 0.19, p = 0.067, η_p_^2^ = 0.01). Magnitude-based inferences showed that, when compared to Pl, CHO ingestion effect is likely negative (94%, CI: −6.1/17.9 ms) on SD pre-sprint and likely positive during exercise (84%, CI: −26.6/6.2 ms). 

No effect from nutritional supplementation and exercise was observed on errors (respectively F_(1,16)_ = 0.27, p = 0.061, η_p_^2^ = 0.02 and F_(1,16)_ = 2.63, p = 0.013, η_p_^2^ = 0.14). Magnitude-based inferences showed that exercise has a likely positive effect (90%, CI: −2.6/0.7%) on accuracy on both conditions. 

## 4. Discussion

The present study aimed to investigate the effect of CHO ingestion on muscular and cognitive performances using multiple sprints exercise with incomplete recovery (6 × 5 s sprints with 25 s of passive recovery). Using a qualitative approach [30], the present findings indicated that CHO ingestion enhances both sprint and cognitive performances. Carbohydrate supplementation increased maximal power output and reduced muscular tiredness compared to Pl ingestion. The results also demonstrated a positive effect of intermittent heavy exercise on all cognitive tasks, manifested by faster information processing and a better efficiency of attention/perception and inhibitory control processes. When compared to Pl, magnitude-based inferences showed that CHO ingestion induced a noticeable positive effect on SRT performances after exercise and on Go/Nogo and visual scanning performances during exercise. 

In the scientific literature, few studies examined the effect of CHO ingestion on sprint performance. Moreover, it is difficult to interpret current results considering their disparity. The complex nature of intermittent sports as well as many methodological differences are most likely responsible for such discrepancy. The improvement in muscular performances observed in the present study argues in favor of an ergogenic effect of CHO, in line with previous results indicating an increase in maximal power output after CHO ingestion [16] or mouth rinse [31,32]. Lee et al. [16] have observed that CHO ingestion has a small but significant effect on repetition sprint exercise performance in female athletes during 10 sets of 5 × 4 s with 20 s recovery. More recent studies have used mouth rinsing to test the central nervous system hypothesis of CHO effect. For example, Beaven et al. [31] have reported an increase in power production during a repetition of 5 × 6-s cycling sprints interspersed with 24 s of active recovery in males cyclists. Recently, Philips et al. [32] have investigated the influence of serial administration of CHO mouth rinse on cycling performance, nausea scales and perceived exertion during a 30 s cycle sprint in physically active males. Using a qualitative approach, they reported a likely benefit of the CHO mouth rinse on maximal power output during a cycle sprint, especially during the first 5 s of the sprint, without any effects on fatigue index or perceived exertion. The present results confirm the positive effect of CHO supplementation on peak power and power maintenance among sprints on a highly trained population. In the field of elite sport, these findings open a new perspective on performance optimization strategy in order to cope with fatigue. 

The second main finding of this study is the effect of CHO ingestion on cognitive functioning after a series of shorter but high intermittent exercises. It is now widely accepted that exercise has a positive effect on cognitive performance and leads to increased arousal [33]. Exercise facilitates multiple cognitive performances and, under certain conditions, enhances responses speed and response accuracy, and facilitates cognitive processes. Most studies focused on the effects of moderate intensity exercise, but results on cognition during heavy exercise are fairly equivocal (for review see [34]). It appears that a positive effect is mainly observed after or during aerobic exercise with a minimum duration of approximately 20 to 60 min [33]. The hypothesis supporting these results is that exercise acts as a stressor that induces changes in arousal level and as a result impacts cognitive functioning. In the acute exercise-cognition interaction, the effect of exercise seems to be highly correlated with the plasma adrenaline (epinephrine) and noradrenaline (norepinephrine) threshold [35]. For example, it has been reported a link between a high-level of blood adrenaline that could be associated with changes in the central nervous system and improvement in cognitive performance [36]. More recently, Dietrich and Audiffren [23] proposed a neurocognitive model to account for the psychological consequences of acute exercise. This model called reticular-activating hypofrontality model, predicts that exercise engages arousal mechanisms in the reticular-activating system and disengages the higher-order functions of the prefrontal cortex with fatigue. However, this model is still matter of debate [37,38]. Recent studies suggest that cognitive control is extremely robust and appears not to be affected by the heavy exercise. For example, Davranche et al. [37] investigated whether workload intensity modulates exercise-induced effect on RT performances. Fourteen participants performed a Simon task while cycling 20 min at a light (first ventilatory threshold, VT − 20%), moderate (VT1) or very hard (VT + 20%) level of exercise. After 15 min of cycling, RT was faster than during the first 5 min of exercise, and any signs of worsening on RT or on accuracy have been reported during heavy exercise. More interestingly, the inhibitory control was fully efficient from the beginning to the end of exercise, regardless of the workload intensity. The results of the present study are in line with these findings, since a positive effect of exercise has been observed on response speed for SRT, visual scanning and Go/Nogo performances after sprint repetition, and suggest that a series of shorter heavy intermittent exercises could enhance cognitive functioning.

The effect of CHO supplementation on cognitive function during exercise has been occasionally assessed, but mainly during endurance exercise. Lieberman et al., 2002 [26] reported that administration of supplemental carbohydrate significantly enhances vigilance and mood during sustained physical activity and interspersed rest. Collardeau et al. [22] have shown that, when compared to placebo, drinking a 5.5% carbohydrate solution improves complex cognitive performance during a two-hour run performed at 75% VO_2max_ by well-trained triathletes. These studies suggest that there is an increase in resource allocation with CHO ingestion during prolonged exercise. Carbohydrate ingestion during exercise possibly leads to a decrease in mental load associated with the perception of effort (RPE) and consequently lead to a higher involvement in attentional resources during the sustained attention tasks [39]. Indeed the role of CHO ingestion on RPE has been widely studied and an improvement in RPE (lower scores) is classically reported. 

There are limited data regarding the effect of CHO ingestion on cognitive function in intermittent sports and the results are conflicting. To date, Bottoms et al. [25] are the only authors who have reported an improvement in visual RT after CHO intake during short duration exercise performed by squash players. Despite the fact that their protocol is far closer to the demands of squash play than the present one, the present findings are in line and support the beneficial effect of CHO ingestion on sprint performance. Welsh et al. [17] and Winnick et al. [18] tested the effect of CHO ingestion with basketball players on an intermittent high-intensity protocol. The authors reported a benefit of CHO ingestion on exercise capacity, but no effects on attention and executive control (e.g., Stroop task) were found.

The present results showed that exercise improves cognitive performances. Furthermore, a positive additional effect of CHO ingestion has been found on information processing during the SRT task and on attention/perceptual performance during the visual scanning. However, no significant changes have been observed on inhibitory control during the Go/Nogo task. One specificity of our design is that the SRT task was performed after sprint, at rest, contrary to VS and GNG which were assessed after sprints but during a cycling exercise. According to Lambourne and Tomporowski [33], deterioration of perceptual responses and SRT has been shown when cognitive tasks are performed in double task. The effect of double tasks (exercise + cognitive task) seems to be more important on simple reaction time than other cognitive tasks.

Two mechanisms have emerged to explain the ergogenic effect of CHO ingestion. The first mechanism, which appears during prolonged exercise lasting more than 1.5 h, is a metabolic effect of CHO oxidation when skeletal muscle and liver glycogen stores are a limiting factor [40]. For shorter exercise, lasting one hour or less, the explanatory mechanism is of central origin in response to exposure of CHO in the oral cavity. Considering the brief duration of the physical task used in this protocol, the facilitating effect cannot be explained by a metabolic effect. We assume that during ingestion, CHO may exert its effects on exercise through a central action, improving motor drive or motivation, mediated by receptors in the mouth or GI tract [40]. The mouth receptors that are sensitive to CHO could have activated specific brain areas, like the orbitofrontal cortex, and elicit beneficial effects on muscular and cognitive performances [10]. 

Numerous studies over the past decade emphasized the potential ergogenic effects of dietary constituents on cognitive performance (for review see [3]). In line with the present findings, it has been suggested that nutritional supplementation such as caffeine [41,42,43], guarana [44,45], tyrosine in a warm environment [46], branched-chain amino acids [47], and nitrate [48] could help to limit central fatigue and increase or maintain cognitive abilities. These findings open a new perspective in terms of performance optimization strategy to cope with fatigue in the field of elite sport. However, more studies are required to complement these results.

The main limitation of the present study is that participants took part in the study in a post-pradial state but no blood glucose baseline levels were measured before experimental session. The fact that the initial carbohydrate status of the athlete was not measured does not question the effects of CHO observed in this study, but makes the generalization of the present findings difficult. Indeed, the blood level of glucose is an important factor since CHO ingestion seems to have a greater impact on performance under circumstances eliciting hypoglycemia. The influence of a CHO intake on performance may be reduced when participants are fed [49,50], therefore the ergogenic effect of CHO ingestion may have been larger if participants had fasted longer than three hours. However several observations indicate that the nutritional status of participants prior testing is not a definitive regulator of the effect of carbohydrate. Whitham and McKinney [51] found no effect of CHO MR on 60-min time trial performance despite an overnight fast, whereas Pottier et al. [52] found a benefit when a high-CHO meal was ingested two hours prior to testing. While the present study involved a relatively small sample size due to the specificity of the population, future studies involving larger sample size are needed to confirm the present results and further elucidate the impact of CHO ingestion on cognitive performance specific to an intermittent sport model.

## 5. Conclusions

To summarize, the present data suggests that CHO ingestion enhances physical performance in high-level squash and fencing athletes during an intermittent high-intensity exercise, and has an additional positive effect on cognitive performances after exercise. Note that, the specificity of our high-level athletes can influence these results as it is generally assumed that attentional breadth continued to increase with higher physical exercise in contrast with non-athletes [53]. 

## Figures and Tables

**Table 1:** Anthropometrical and physiological characteristics of participants.

Variables	All Mean ± Standard Error	Squash Athletes	Fencing Athletes
Sample size	17	8	9
Age (years)	19.1 ± 1	20.8 ± 2	17.3 ± 0.3
Height (cm)	170.6 ± 2	174.5 ± 4	167.1 ± 3
Body mass (kg)	63.9 ± 3	67.1± 5	61.1 ± 2
Body fat (%)	15.3 ± 1	11.9 ± 1	18.4 ± 2
VO_2max_ (mL·min^−1^·kg^−1^)	48.5 ± 2	50.7 ± 1	47.5 ± 4
Heart rate_max_ (batt·min^−1^)	200.8 ± 2	199.2 ± 2	202 ± 3
Blood lactate post test (mmol·L^−1^)	12.7 ± 1	12.7 ± 1	12.8 ± 1
Maximal aerobic speed (km·h^−1^)	14.5 ± 0.4	16.2 ± 0.4	14.5 ± 0.5

**Table 2:** Cognitive performance prior and after sprints repetition for Pl and CHO ingestion.

Variables	Prior sprints		After sprints	
	Pl Mean ± Standard Error	CHO	Pl	CHO
SRT			*At rest*	
RT (ms)	222.5 ± 9	229.0 ± 8	220.4 ± 8 ^$^	203.1 ± 5 ^$,£,#,^*
SD (ms)	88.6 ± 22	67.0 ± 15 ^£^	74.8 ± 12	108.9 ± 22 ^£^
Visual Scanning			*During cycling exercise*	
RT (ms)	2010.3 ± 107	2054.1 ± 133	1726.8 ± 73 ^$,^*	1630.6 ± 76 ^$,£,^*
SD (ms)	867.3 ± 78	829.0 ± 75 ^£^	689.6 ± 49 ^$,^*	665.2 ± 60 ^$,^*
Tiredness (%)	3.8 ± 3	5.5 ± 2	1.5 ± 2 ^$^	−0.4 ± 2 ^$^
Errors (%)	4.8 ± 1	5.9 ± 2 ^£^	7.1 ± 1 ^$,^*	7.8 ± 1 ^$,^*
Go/Nogo			*During cycling exercise*	
RT (ms)	444.1 ± 17	453.1 ± 13	453.1 ± 18 ^$^	435.4 ± 12 ^$,£^
SD (ms)	84.2 ± 7	88.9 ± 9 ^£^	90.1 ± 9	79.1± 8 ^£^
Errors (%)	6.3 ± 2	5.3 ± 1	2.6 ± 1 ^$^	4.9 ± 1 ^$^

Notes: * quantitative statistics shown an exercise effect (Pre sprints _Pl/CHO_ vs. Post sprints _Pl/CHO_); ^#^ quantitative statistics shown a nutritional effect (Pl_pre/post_ vs. CHO_pre/post_); ^$^ qualitative statistics shown an exercise effect (Pre sprints _Pl/CHO_ vs. Post sprints _Pl/CHO_); ^£^ qualitative statistics shown a nutritional effect (Pl_pre/post_ vs. CHO_pre/post_).
